# Thyroid Cancer Surgery in a Patient With Frontotemporal Dementia: Balancing Oncologic and Neurocognitive Challenges

**DOI:** 10.7759/cureus.91609

**Published:** 2025-09-04

**Authors:** Anastasios Bontozis, Alexandros Makris, Nikolaos Tzanakis, Christos Politopoulos, Georgios Giannopoulos

**Affiliations:** 1 Department of Anesthesiology, Asklepieion Voulas General Hospital, Athens, GRC; 2 Department of Surgery, Asklepieion Voulas General Hospital, Athens, GRC

**Keywords:** anesthetic risk, bis monitoring, frontotemporal dementia, papillary carcinoma, thyroid cancer

## Abstract

We describe the perioperative management of a patient with severe frontotemporal dementia (FTD) who presented with progressive dysphagia due to a large compressive thyroid mass. Despite a non-diagnostic fine needle aspiration (FNA - Bethesda I), a left lobectomy and isthmectomy were performed to relieve symptoms while minimizing risk of recurrent laryngeal nerve injury and reducing operative time. The patient had a poor neurological prognosis, severe cognitive impairment, and increased anesthetic risk. Airway management was challenging due to tracheal deviation and compression, limited neck mobility, and suspected oropharyngeal reflex impairment. Intubation was successfully performed using a videolaryngoscope and a gum elastic bougie, followed by placement of an armored orotracheal tube. Anesthesia was maintained with low-dose propofol administered via continuous infusion, guided by bispectral index (BIS) and clinical signs. BIS values remained low throughout the procedure. The patient was extubated safely and recovered uneventfully. Pathology revealed papillary thyroid cancer.

## Introduction

Frontotemporal dementia (FTD) is a common cause of early-onset neurodegenerative disease, characterized by progressive impairment in behavior, language, and executive function [1]. In advanced stages, FTD can lead to autonomic dysfunction, altered airway reflexes, and reduced patient cooperation, significantly complicating perioperative care [2].

Large thyroid masses, often exhibiting cystic degeneration, may pose diagnostic challenges with fine needle aspiration (FNA), particularly when cytology yields non-diagnostic results. When significantly enlarged, these lesions can cause compressive symptoms, such as dysphagia, dyspnea, or tracheal deviation, and in such cases, surgical intervention is frequently required. The choice of procedure, ranging from lobectomy to total thyroidectomy, must balance the relief of symptoms and oncologic considerations against the risks of recurrent laryngeal nerve (RLN) injury and prolonged anesthesia.

The extent of surgery is also a dilemma. Each side of thyroidectomy carries its own independent risk of RLN injury, especially in cases like this, let alone the prolongation of general anesthesia. Larynx function and breathing ability are very fragile, so is the delicate balance between successful airway management and extubation risk.

The coexistence of severe FTD and a large compressive thyroid mass presents a complex clinical scenario, requiring surgical planning that accounts for both anatomical challenges and the patient’s overall prognosis. This case underscores the importance of multidisciplinary, individualized decision-making when managing surgical pathology in the context of advanced neurodegeneration. It also raises ethical considerations emphasizing symptom relief and safety rather than oncologic completeness.

## Case presentation

A 65-year-old male patient was referred to our endocrine surgeon for progressive dysphagia and a palpable left-sided thyroid mass. His medical history was significant for FTD, for which he was under regular follow-up. His treatment included amisulpride and mirtazapine. Neurological assessment revealed a baseline state of alertness with limited awareness of external stimuli and partial recognition of basic needs, such as eating, drinking, and self-care. The patient exhibited Parkinsonian features and was classified as having severe FTD, not able to complete the mini-mental state examination due to the absence of meaningful verbal output (score: 0/30).

His family noticed an enlarging neck mass, initially evaluated with a neck ultrasound. Examination revealed a 6 cm, heterogeneous left thyroid mass with cystic elements. The right thyroid lobe was unremarkable. FNA was reported as Bethesda I, indicating non-diagnostic cytology due to insufficient cellularity and therefore not excluding malignancy.

Given the neurological status, his kin were reluctant to undergo any surgical intervention. In an attempt to relieve symptoms as the mass was growing bigger, the patient underwent an ultrasound-guided aspiration of the cystic nodule, but the procedure was abandoned due to bleeding, which was managed conservatively.

**Figure 1:**
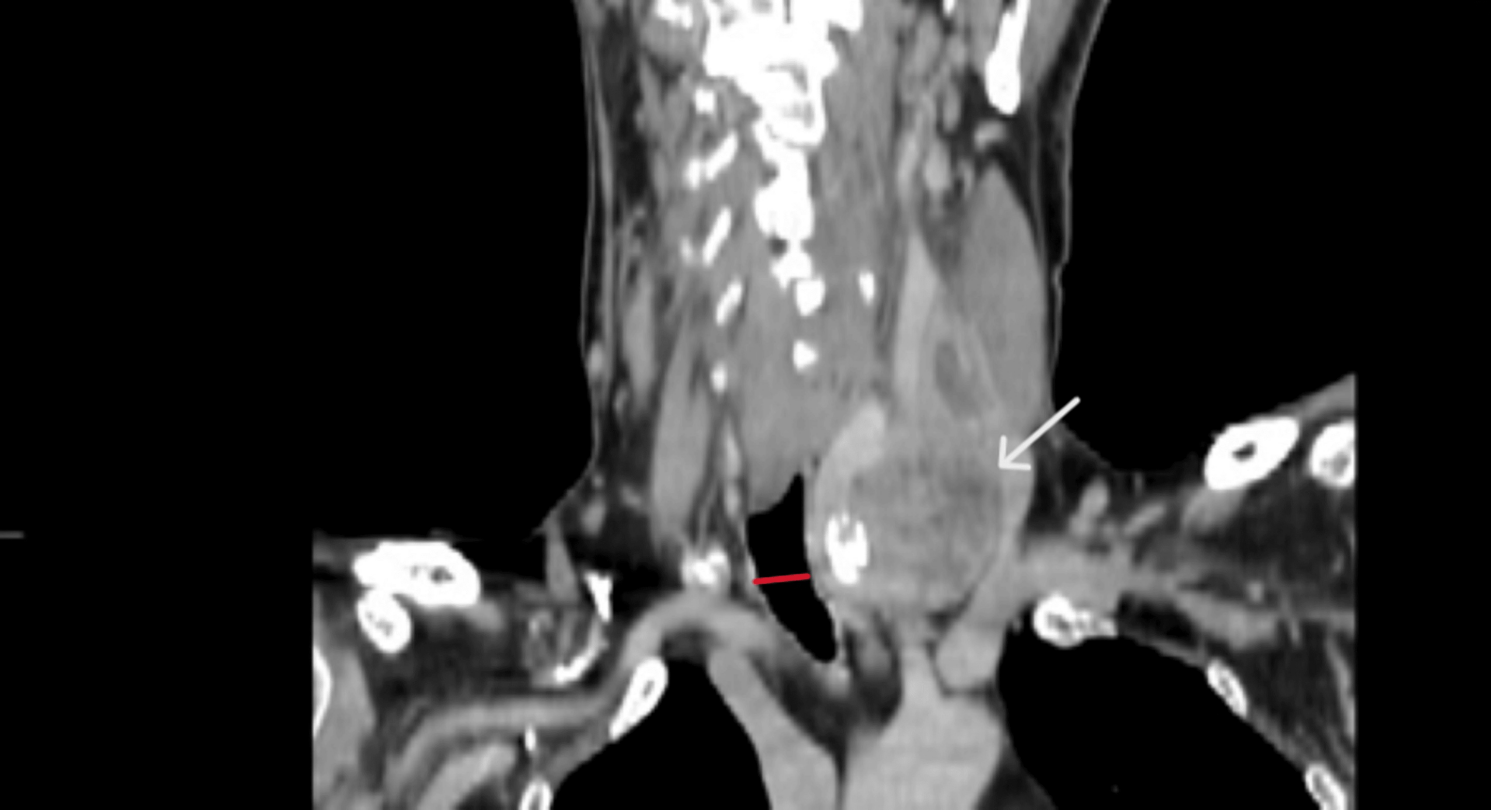
CT - coronal. Tumor with cystic element (white arrow) and calcification, compressing the carotid artery and jugular vein. The narrowed tracheal segment is 1.27 cm in diameter (red line).

**Figure 2:**
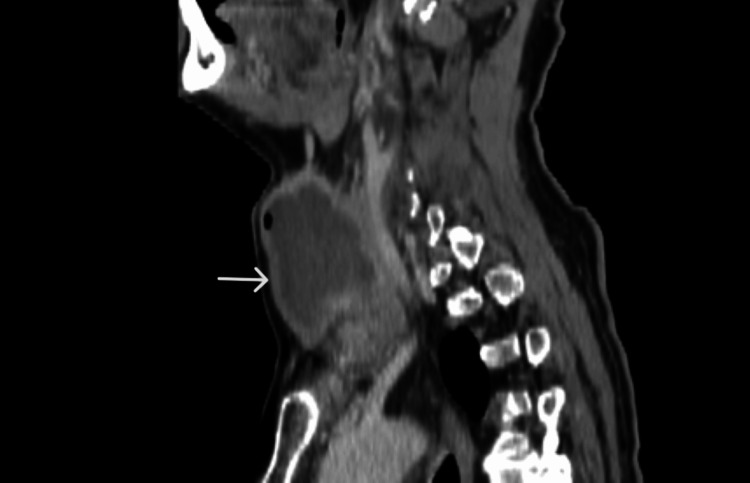
Neck CT - sagittal view. Cystic degeneration is prominent (white arrow).

**Figure 3:**
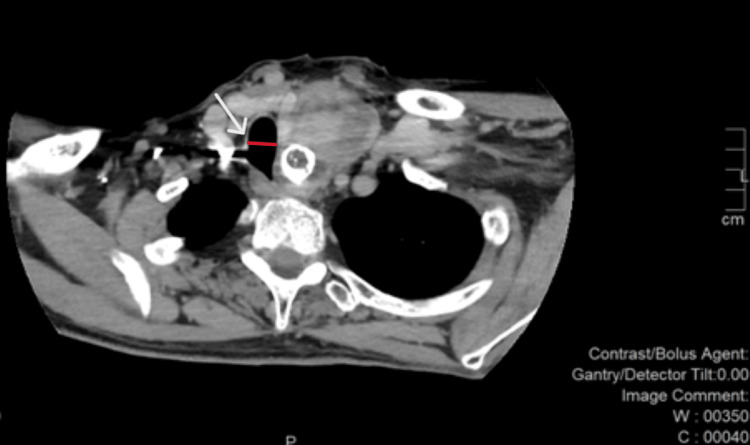
Neck CT - axial view. Tumor calcification compressing and deviating the trachea. The narrowed tracheal segment measures 1.27 cm in diameter, as indicated by the white arrow and the red line.

**Figure 4:**
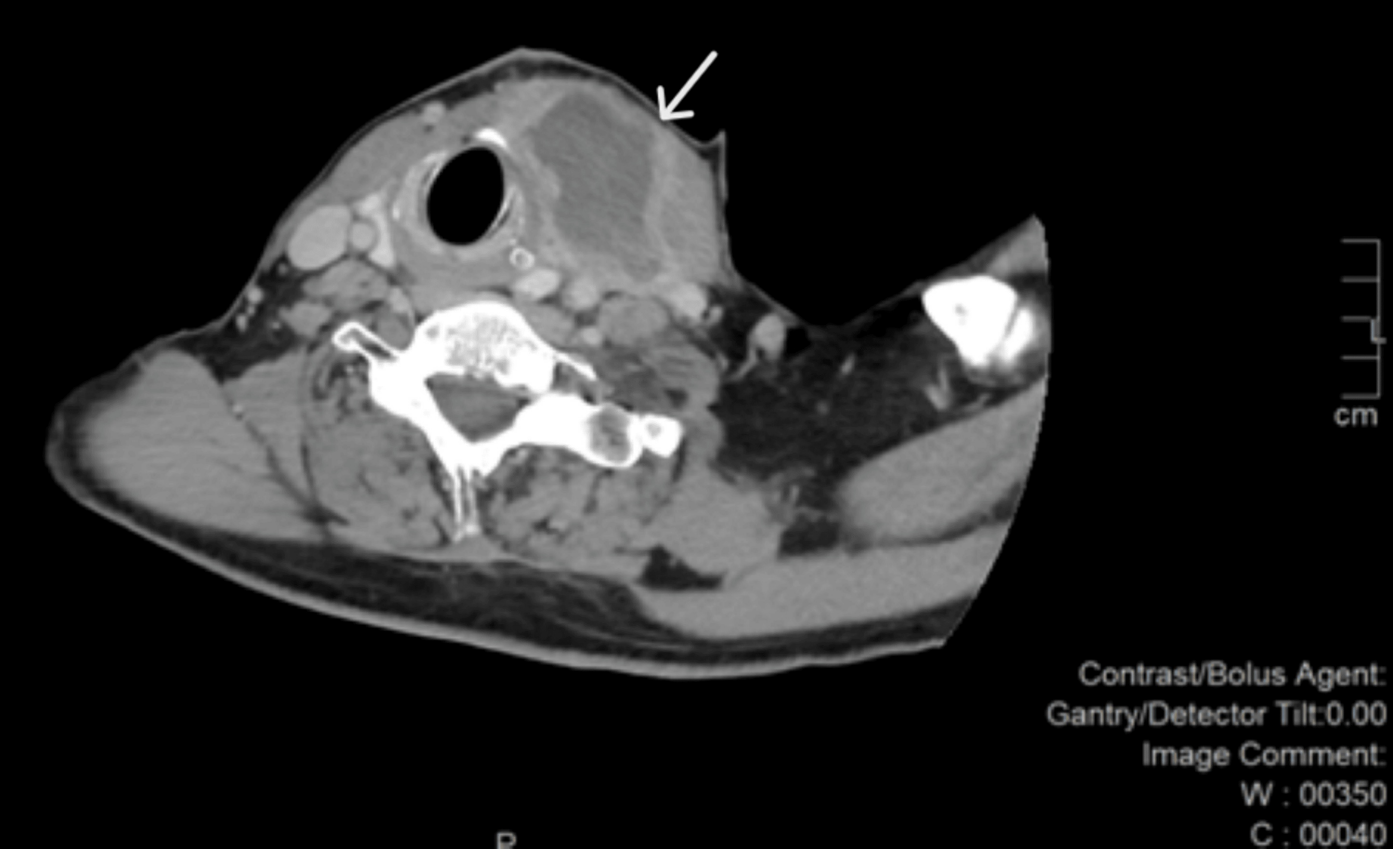
Neck CT - axial view: part of the tumor with cystic degeneration (white arrow).

Subsequent neck computed tomography demonstrated significant tracheal compression, with a narrowed segment measuring 1.27 cm in diameter (Figures 1-4).

In view of the rapid tumor progression and ongoing symptoms, surgical intervention was recommended without repeating the FNA. The patient’s legal representatives provided consent after acknowledging the risks and benefits of the procedure.

Preoperative laboratory results, including thyroid function tests, were within normal limits. The patient was euthyroid. Cardiac evaluation revealed only an incomplete right bundle branch block (RBBB).

A plan was formulated for tracheal intubation using videolaryngoscopy to optimize visualization of the glottis and minimize airway trauma. A gum elastic bougie was prepared as an adjunct to facilitate tube placement in case of restricted view, and an armored, flexible, orotracheal tube (size 8.0) was selected to reduce the risk of kinking or obstruction during surgery. A backup strategy with alternative airway devices (supraglottic airway, fiberoptic bronchoscope) and surgical airway equipment was also prepared. In theatre, standard monitoring was established, including two 20-G peripheral IV lines, neuromuscular blockade monitoring, and bispectral index (BIS) monitoring. General anesthesia was induced with intravenous administration of 200 mg propofol, 100 µg fentanyl, and 50 mg rocuronium. Tracheal intubation was achieved uneventfully with videolaryngoscopy and bougie guidance, followed by insertion of the armored tube. Anesthesia was maintained with a continuous low-dose propofol infusion titrated between 2 and 3 mg/kg/h. The rate was sparsely adjusted in 0.5 mg/kg/h steps in response to BIS trends, raw EEG, and additionally clinical signs (absence of motor/autonomic response to surgical stimuli), as BIS values remained low throughout despite light infusion rates [3]. Intraoperatively, after the initial fentanyl dose was administered at induction, the patient received an additional 150 µg during the course of surgery, in combination with 1 g of intravenous paracetamol.

The patient remained hemodynamically stable throughout most of the procedure. One episode of hypotension was recorded 45 minutes after the induction of anesthesia, as a potential sign of autonomic instability, with blood pressure dropping to 75/43 mmHg and heart rate increasing to 105 bpm, which was effectively managed with bolus doses of phenylephrine. Initial vital signs were 135/60 mmHg and 81 bpm.

**Figure 5:**
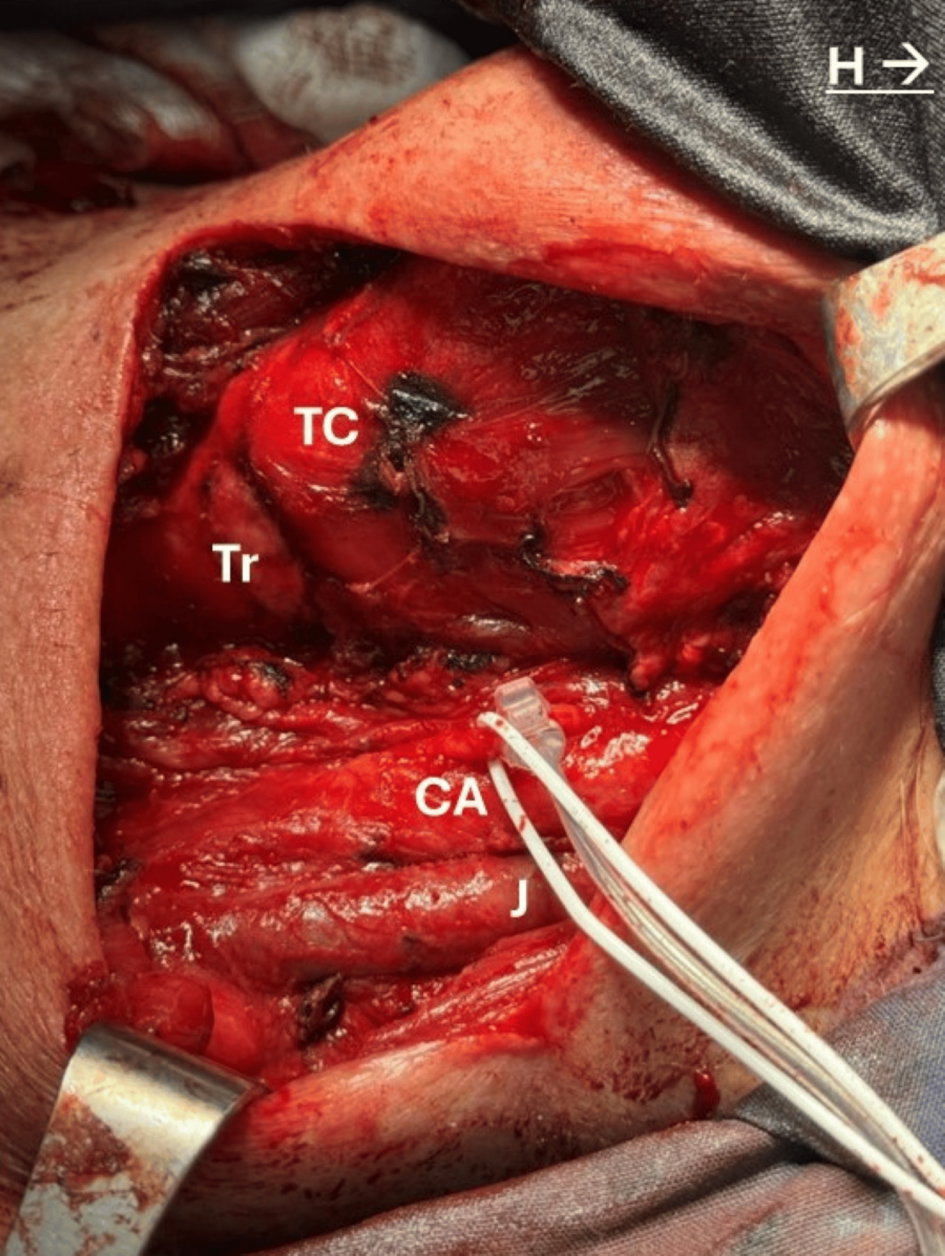
Operative field after tumor resection. White vesseloop around the vagus nerve with electrode for continuous monitoring. TC: thyroid cartilage, Tr: trachea, CA: carotid artery, J: jugular vein, H: head

At the time of surgery, the left thyroid lobe was massive, compressing both the trachea and internal jugular vein (Figure 5).

The carotid artery and RLN were also dislocated. Nevertheless, none of these structures was directly invaded. Continuous monitoring of the vagus nerve (VN) was established and evaluated according to the International Neuromonitoring Study Group (INMSG) guidelines [4]. Left RLN was dissected from the tumor, without alterations in the VN signal.

Due to unclear FNA results, grossly normal right lobe, high anesthetic risk, and poor neurological prognosis, it was deemed safer to perform a left lobectomy and isthmectomy. Removal of the mass was completed uneventfully, maintaining normal VN function throughout surgery.

**Figure 6:**
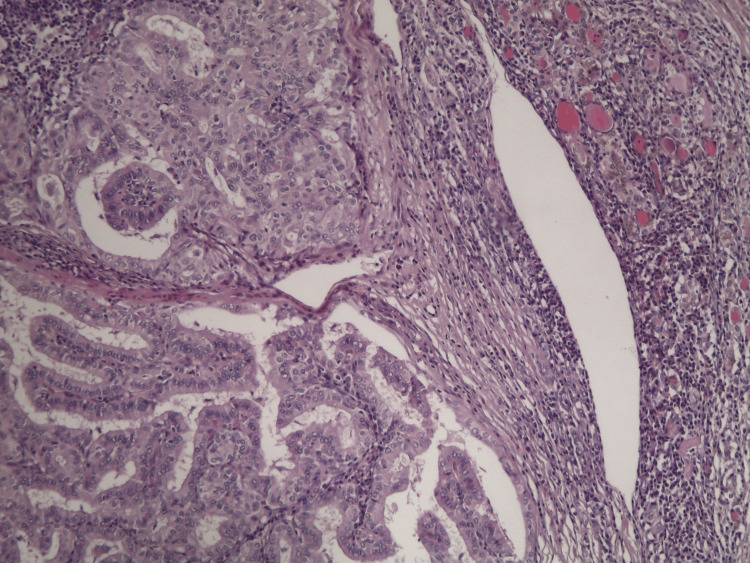
Papillary carcinoma with complex, arborizing and delicate papillary structures (hematoxylin-eosin stain, ×100 magnification).

The patient regained consciousness and was extubated in theatre, maintaining stable vital signs. The duration of general anesthesia was two hours and 35 minutes, while the operative time was two hours and 10 minutes. For postoperative analgesia, a regimen of scheduled paracetamol 1 g four times daily was administered, with tramadol provided on an as-needed basis. Pain control was effective, and no opioid-related complications were observed. Eventually, he was discharged the next day. Pathology reported a PT3a papillary carcinoma with clear margins (Figure 6).

**Figure 7:**
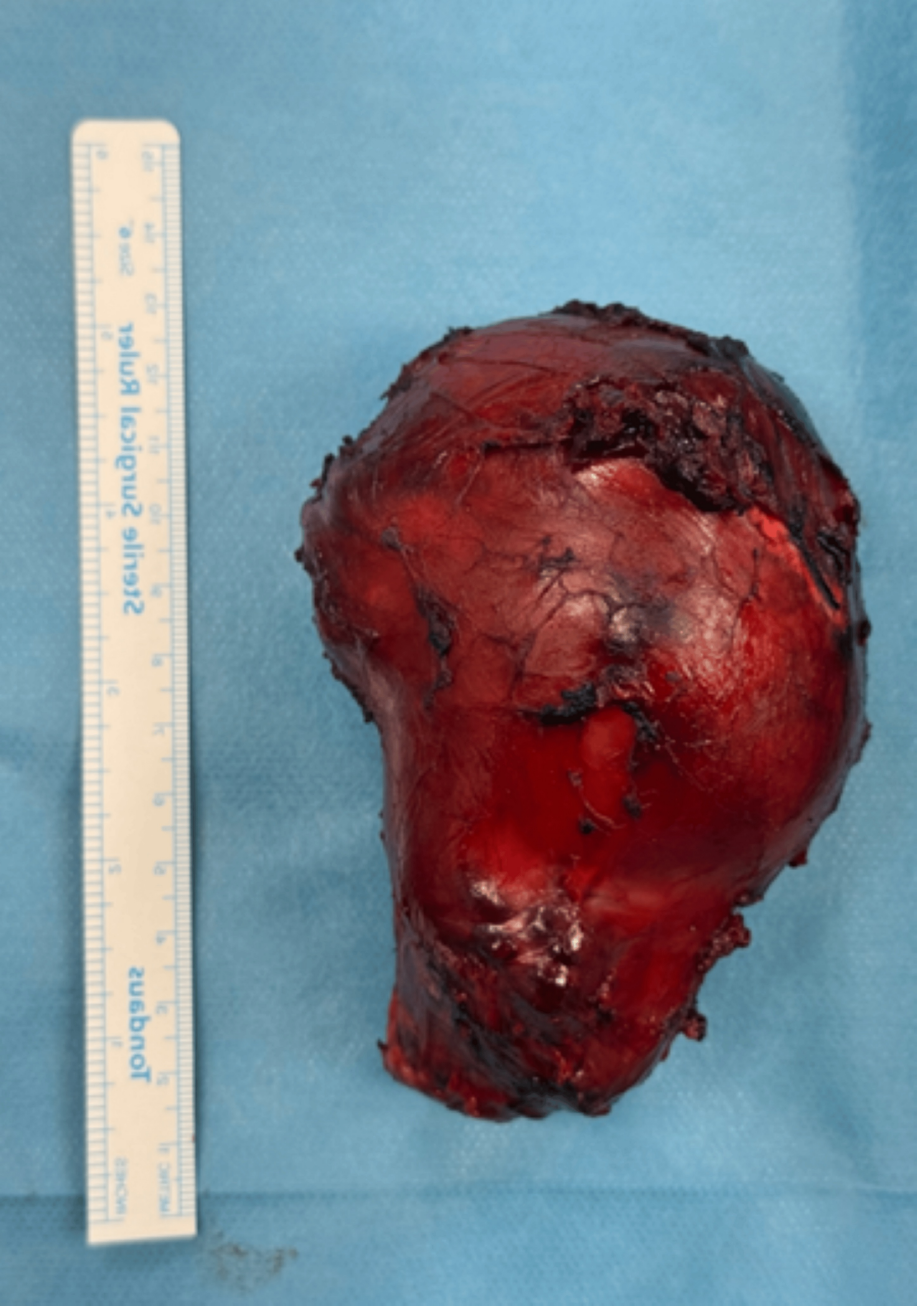
Specimen after excision, measuring 12.5 cm in maximum diameter.

The specimen weighed 128 g, with a maximum diameter of 12.5 cm (Figure 7).

The patient recovered uneventfully and returned to baseline neurological function, with no new deficits observed in the immediate postoperative period. In the first review after three months, his family described his swallowing capability as improved. A new neck ultrasound showed no sign of tumor recurrence. After thorough discussion, it was decided that a complete thyroidectomy would pose a significant risk, so surveillance with TSH suppressive levothyroxine was chosen instead. The surveillance plan comprised TSH suppression, periodic clinical review, and neck ultrasonography at 6-12 month intervals.

## Discussion

The case was managed in a tertiary referral hospital by an experienced endocrine surgeon and a senior anesthesiologist, both familiar with thyroid surgery and neuromonitoring. Preoperative planning was multidisciplinary, involving surgical, anesthetic, and neurological input. The setting provided full neuromonitoring and critical care support.

FTD is a progressive neurodegenerative disorder that primarily affects the frontal and temporal lobes of the brain, which are responsible for personality, behavior, and language. It is one of the most common causes of early-onset dementia, typically emerging in midlife. FTD leads to significant changes in social conduct, emotional regulation, and communication, placing a considerable burden on patients and their caregivers [5].

The patient experienced intraoperative hypotension with reflex tachycardia in the absence of blood loss or anesthetic changes. This may reflect impaired autonomic regulation, a feature described in advanced FTD. The disease affects several cortical areas, namely, the orbitofrontal cortex, the frontal medial cortex (including the anterior cingulate), the insula, andthe cerebral amygdala, with the insular cortex playing a particularly important role given its integration of many autonomic functions. Although rare, FTD-associated severe autonomic dysregulation has been reported, manifesting as unpredictable cardiovascular responses under anesthesia, including exaggerated sensitivity to vasodilatory effects and blunted baroreceptor reflexes [2].

In this patient, BIS values remained in a range typically indicative of deep anesthesia, despite low-dose propofol infusion. Clinically, however, the patient was appropriately anesthetized [3]. Interestingly, upon emergence from anesthesia, BIS values remained low, even though the patient was clearly awake and able to be extubated. This observation is likely attributable to the underlying FTD. Several studies have demonstrated that processed EEG indices, such as BIS, may not accurately reflect brain states in patients with FTD or Alzheimer’s disease, as the neurodegenerative changes can significantly attenuate baseline cortical rhythms [6].

Airway management followed a structured plan as mentioned above, due to a narrowed, deviated trachea and reduced cervical mobility. Furthermore, impairment of oropharyngeal reflexes was suspected [7]. These anatomical and functional challenges necessitated a carefully planned and optimized approach to airway management to ensure safe and controlled intubation.

Surgical strategy was restricted to symptomatic relief, regardless of cytology. The patient had a severely impaired level of awareness, with only partial recognition of basic needs such as feeding and self-care [8]. Given the patient's overall frailty and limited prognosis, a bilateral, prolonged procedure was considered perilous [9].

Nevertheless, the rationale of lobectomy has been clearly demonstrated in the INMSG guidelines. Bilateral operation means “two nerves at risk,” and in this case, even a mild, unilateral RLN paresis could have detrimental effects on the outcome. A lobectomy with isthmectomy provided resolution of compressive symptoms while avoiding the prolonged anesthesia and higher risk of bilateral nerve injury associated with total thyroidectomy [4]. Left lobectomy, though challenging, was completed with no impact on RNL/VN function, and the right side could be safely visited in the future, if necessary [10].

Due to the complexity of the case and the uncertain outcome, an intensive care unit (ICU) bed had been secured, although not used. All the above reflect the importance of a holistic, patient-centered approach in high-risk individuals with limited functional reserve [11].

## Conclusions

In selected high-risk patients with poor prognosis, a more conservative or staged thyroid surgery may alleviate symptoms while minimizing risk. ICU readiness should be considered in complex surgical cases involving cognitive and airway compromise. Additionally, BIS monitoring may underestimate consciousness levels in patients with frontotemporal dementia due to altered cortical EEG activity, and the autonomic dysfunction in this type of dementia needs careful perioperative planning. Thus, this case highlights the value of careful multidisciplinary planning and readiness for hemodynamic and airway instability in high-risk patients with severe neurocognitive impairment.
